# Safety and efficacy of Jujadokseo-hwan for memory deficit (amnesia) in mild neurocognitive disorder: A protocol for randomized, double-blind, placebo-controlled, parallel-group, multicenter clinical trial

**DOI:** 10.1097/MD.0000000000019231

**Published:** 2020-02-21

**Authors:** Jin-Hyung Jeong, Ji-Yoon Lee, Ju-Yeon Kim, Young-Kyung Seo, Wee-Chang Kang, Hyung-Won Kang, So-Jung Park, Hye-Kyoung Jang, Yang-Chun Park, In Chul Jung

**Affiliations:** a Department of Oriental Neuropsychiatry, College of Korean Medicine; b Department of Statistics, H-Liberal Arts College, Daejeon University, Daejeon; c Department of Korean Neuropsychiatry Medicine & Inam Neuroscience Research Center, Wonkwang University Sanbon Hospital, Gyeonggi-do; d Clinical Trial Center, Dunsan Korean Medicine Hospital of Daejeon University, Daejeon, Republic of Korea.

**Keywords:** herbal medicine, Jujadokseo-hwan, memory deficit, mild cognitive impairment, mild neurocognitive disorder

## Abstract

**Background:**

Mild neurocognitive disorder (mNCD) is one of the degenerative diseases that results in memory deficit, and can progress to dementia. The effectiveness of drug therapy for mNCD is still debatable, but treatment of this disease has important implications for postponing or preventing dementia. Jujadokseo-hwan (JDH) is a traditional herbal medicine formulation that exhibits improvement in cognitive abilities and neuroprotective effects. In this study, we will evaluate the safety and efficacy of JDH compared to placebo in mNCD patients.

**Methods and design:**

This is a randomized, double-blind, placebo-controlled, parallel-group, multicenter clinical trial. After obtaining informed consent from all participants and performing the screening process, the participants will be equally divided into an experimental group and a control group. Each participant will visit the trial center 7 times during the 12 weeks of intervention. The follow up study will be conducted 12 weeks after the intervention ends. The primary outcome is the variance in Seoul verbal learning test-elderly's version (SVLT-E) score from baseline to 12 weeks. Secondary outcomes include scores/values for the following variables: SVLT-E, Rey complex figure test, Digit span test, Korean-Boston naming test, calculation ability, controlled oral word association test, Korean-color word stroop test, digit symbol coding, Korean-trail making test-elderly's version, Korean version of mini mental state examination for dementia screening, euro-qol-5 dimension, pattern identifications tool for cognitive disorders, Korean version of Montreal cognitive assessment, Korean quality of life-Alzheimer disease, computerized tongue image analysis system, blood pressure pulse analyzer, bioelectrical impedance analyzer, wearable electroencephalogram device, functional near-infrared spectroscopy system, and cost analysis.

**Discussion:**

This is the first trial evaluating the efficacy of JDH for mNCD. We expect this trial will provide strong support for wide use of JDH for mNCD and lead to further research on herbal medicine treatments for mNCD.

**Trial registration number:**

KCT0003570 (Registered in Clinical Research Information Service of the Republic of Korea, https://cris.nih.go.kr/cris/search/search_result_st01.jsp?seq=12669).

## 1 Introduction

Recently, degenerative diseases have a higher prevalence due to extended life expectancies. Among them, dementia has the fourth highest mortality rate after cancer, heart disease, and stroke.

Currently, mild neurocognitive disorder (mNCD) is known as a precursor for dementia.^[1]^ However, there is no apparent cure and a variety of alternative treatments are being evaluated.^[2,3]^ Various clinical studies have reported using herbal medicines to improve memory and cognition, although no well-designed randomized controlled trials have been performed so far.^[4–6]^

Jujadokseo-hwan (JDH, ZhuziDushu Wan in Chinese) is a traditional herbal medicine formulation listed in Uihak-ipmun (Yixue Rumen in Chinese). It is composed of 7 herbal components: Acori Graminei Rhizoma, Angelica Gigantis Radix, Citri Unshius Pericarpium, Ginseng Radix, Glycyrrhizae Radix et Rhizoma, Poria Sclerotium, and Poly galae Radix. In South Korea, a tablet form of JDH is approved for memory deficit (amnesia) by the Korean Ministry of Food and Drug Safety. Two experimental studies investigated the effects of JDH on the brain ability and oxidative stress and found that JDH improved brain ability, learning, and memory, and also demonstrated a neuroprotective effect.^[7,8]^

We plan to conduct a randomized, double-blind, placebo-controlled, parallel;-group, multi-central trial to evaluate the safety and efficacy of JDH in patients with mNCD.

## 2 Methods

### 2.1 Objectives

The main objective of this study is to evaluate the safety and efficacy of JDH compared to placebo for treating mNCD.

### 2.2 Study design and setting

This study is designed as a randomized, double-blind, placebo-controlled, parallel-group, multi-center trial. The clinical trial sites are as follows: Dunsan Korean medicine Hospital of Daejeon University, Wonkwang university Sanbon hospital, and Wonkwang university oriental medical hospital, Jeonju. The anticipated number of eligible participants is 80. Subjects will be divided into 2 equal groups: the JDH group (experimental group) and the placebo group (control group). Each group will be administered JDH or placebo 3 times day for 12 weeks (84 days). After the 12-week follow-up period, participants will be instructed to visit the trial center once for safety and efficacy assessments. The primary outcome is the Seoul verbal learning test-elderly version (SVLT-E)^[9]^ score variable. The secondary outcomes include scoring variables from the SVLT-E, Rey complex figure test (RCFT),^[10]^ digit span test (DST),^[11]^ Korean-Boston naming test (K-BNT),^[12]^ calculation ability, controlled oral word association test (COWAT),^[13]^ Korea-Color word stroop test (K-CWST),^[14]^ digit symbol coding (DSC),^[15]^ Korean trail-making test-elderly's version (K-TMT-E),^[16]^ Mini-Mental state examination for dementia screening (MMSE-DS),^[17]^ EuroQol-5 Dimension (EQ-5D),^[18]^ Pattern identifications tool for cognitive disorders (PIT-C),^[19]^ Montreal cognitive assessment-Korean version (MoCA-K),^[20]^ and Korean quality of life-Alzheimer's disease (KQOL-AD) scale.^[21]^ Other secondary outcomes include measured values from computerized tongue image analysis system (CTIS, Device name: TAS-4000), blood pressure pulse analyzer (BPPA, Device name: DMP-life), bioelectrical impedance analyzer (BIA, Device name: InBody S-10), wearable electroencephalogram device (WED, Device name: neuroNicle FX2), functional near-infrared spectroscopy system (fNIRS, Device name: NS1-H20AM) and cost analysis. This protocol (version 2.1, 28 August 2018) follows the Recommendations for Interventional Trials (SPIRIT) Standard Protocol guidelines.

### 2.3 Participants

#### 2.3.1 Recruitment

Recruitment of the participants began in August 2018 and is expected to finish in August 2020. Recruitment may be extended depending on registration completion. All participants will be given a consent form with a full explanation before registration, and will be notified that they may be withdrawn from the trial at any point without penalty. The consent form includes information regarding background, purpose of the study, trial and placebo product, outcome, and expected benefits and harms.

#### 2.3.2 Screening

Participants will be assigned a screening code (ie, DS-S-001) in the order of consent received. After the screening process, selected participants will be assigned an identification code (ie, DS-E-001). Any medication that subjects have taken 4 weeks before participating in the clinical trial may be accepted at the discretion of the researchers, if they are not prohibited drugs. If participants take these drugs during the trial, they will be discontinued from the trial or their data will be excluded from analysis.

#### 2.3.3 Inclusion criteria

(1) Males and females 45 years and older and less than 85 years old
(2) Participants who are diagnosed with mild neurocognitive disorder based on DSM-5 diagnostic criteria
(3) Participants with CDR score of 0.5 and GDS (global deterioration scale) score of 2-3
(4) Participants with MoCA-K score of 22
(5) Participants who have no limitations in activities of daily living (K-IADL score under 0.43)
(6) Participants who have a level of education of more than 6 years (more than elementary education level)
(7) Participants or authorized surrogates who voluntarily sign the clinical trial consent form

##### 2.3.3.1 Exclusion criteria

(1) Patients with Alzheimer disease, vascular dementia, Parkinson disease, Huntington disease, hydrocephalus, and so on.
(2) Patients with other general conditions associated with dementia, such as hypothyroidism, vitamin B12 or folate deficiency, niacin deficiency, hyperkalemia, neurosyphilis, human immunodeficiency virus, and so on.
(3) Patients with a SGDS (short-form geriatric depression scale) score greater than or equal to 10
(4) Patients who have had major psychiatric disorders such as schizophrenia, delusional disorder, bipolar disorder, alcohol or substance abuse disorder, etc. where were diagnosed according to DSM-5 diagnostic criteria
(5) Patients who have had other neurologic diseases such as epilepsy, brain injury, stroke, and so on.
(6) Patients who have been taking anti-dementia drugs such as acetyl cholinesterase inhibitors or hormones
(7) Patients who have gastrointestinal, endocrine, and/or cardiovascular disease not controlled by diet or medication
(8) Patients with unstable medical conditions (investigators will judge will these conditions according to the standard work procedure, based on the results of vital signs, clinical pathology, electrocardiogram, chest radiography, etc)
(9) Patients with type 2 diabetes or insulin-dependent diabetes mellitus not controlled by hypoglycemic drugs
(10) Patients with hypertension as defined by: systolic blood pressure above 160 mm Hg, diastolic blood pressure above 95 mm Hg
(11) Patients who have had clinically significant liver disease or have serum aspartate aminotransferase, alanine aminotransferase, and/or gamma-glutamyl transpeptidase values that are more than twice the laboratory test upper limit reference.
(12) Patients who have had chronic renal failure or have serum creatinine value that are more than one and a half times the laboratory test upper limit reference
(13) Patients who are pregnant, lactating, or not using appropriate methods of contraception
(14) Patients who received Korean medicine treatment for mNCD during the month before the start of the trial within a month before the trial
(15) Participants who have participated in other interventional clinical trials during the month before the start of the trial
(16) Patients who are assessed as ineligible to participate in the trial by the principal investigator for other reasons

### 2.4 Study procedure

The participants who provide written consent and meet the inclusion and exclusion criteria will be selected based on results from the demographic information survey, medical history (past, present, family), vital signs (blood pressure, pulse rate, body temperature), physical examination, laboratory tests, and CDR, GDS, K-IADL, MoCA-K, SGDS scores at the screening visit. Eligible participants will be informed via telephone call and will be asked to visit the trial center within 1 week (visit 1). If participants are taking drugs that may affect the trial, a washout period of at least 15 days will be required before trial start. At visit 1, investigator will check the result from the screening period and changes since that period, and subjects will be allocated to the experimental or the control group using a stratified block randomization method. After randomization, the participants will be administered 6000 mg of JDH or placebo product daily for 12 weeks. Both groups will be instructed to consume the tablets orally, 3 times a day before meals. Subsequently, subjects will undergo assessments for primary and secondary endpoints (SVLT-E, RCFT, DST, Calculation, K-CWST, DSC, K-TMT-E, COWAT, K-BNT, MMSE-DS, MoCA-K, PIT-C, EQ-5D, KQOL-AD, cost analysis, CTIS, BPPA, BIA, WED, fNIRS). They will visit the trial center for evaluation of any changes or adverse effects every 2 weeks. Tests and assessments will be performed according to the following schedule: Visit 2 (Blind test); Visit 4 (WED, fNIRS, MoCA-K, PIT-C, EQ-5D, KQOL-AD); Visit 7 (all tests); and Visit 8 (SVLT-E, Cost analysis, MoCA-K, PIT-C, EQ-5D, KQOL-AD). Detailed assessment schedules are outlined in Table 1. A follow up study will be conducted 12 weeks after the conclusion of the trial.

**Table 1:**
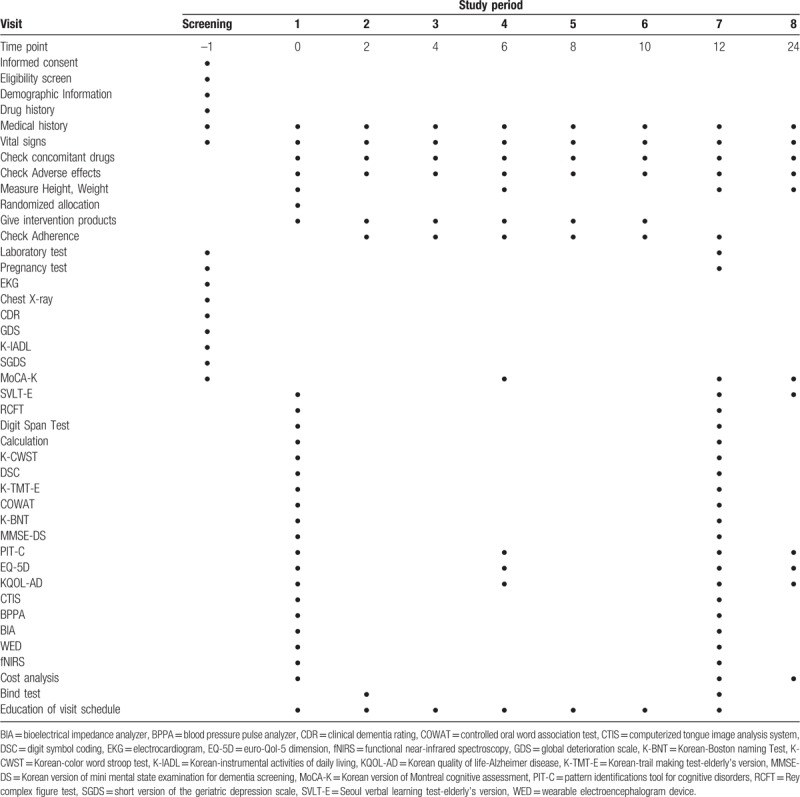
Schedule of trial visits.

### 2.5 Intervention

The shape and appearance of the JDH tablets and the placebo tablets are the same: black-colored herbal tablets. Each tablet is manufactured at a dosage of 100 mg, with shelf-life of 2 years. The manufacturing company (Hanpoong Pharm. Co., Ltd 100, Hyoryeong-ro, Seocho-gu, Seoul, Repubic of Korea) follows the regulations for good clinical practice, and controls the quality of the products using their own standards and testing methods.

The principle investigator (PI) will purchase the products used for the clinical trial from the company and supply them to the management pharmacist. Before labeling, 20 tablets will be put in 1 package to prevent mixing. 60 packages, which includes 42 packages for 14 days plus 18 packages for spare will be prepared at 2 week intervals according to the visit schedule. At visit 1 and 6, 51 packages which includes 42 packages for 14 days plus 18 packages for spare will be given. The product label for clinical trials will be filled out following the Regulation on Safety of Pharmaceuticals, and so on. All products used in the trial will be recorded, including the amount of the medication, the date of the delivery, and the date of return.

After randomization, the participants will begin taking 20 tablets of JDH or the placebo by mouth, 3 times a day before meals, for 12 weeks.

### 2.6 Randomization, blinding

Independent statisticians who are not involved in the trial procedures will generate the random sequence list using an Excel program. A stratified block randomization method will be used for randomized allocation, for which the stratification factors are the trial centers. The randomized code will be kept in opaque sealed envelopes. Randomized code generation and drug blinding will be implemented independent of the data. The pharmaceutical manufacturing company will label the identification code on the products (JDH and placebo) collectively, and hand them to the PI. The PI will let the management pharmacist to be in charge of the products. At the researcher's request, the management pharmacist at the trial center will provide the participants with either JDH or placebo, corresponding to the identification code in a ratio of 1:1. Both the participants and the investigators will be blinded until completion of the trial.

### 2.7 Sample size calculation

Using 80% power for detecting treatment differences, a significance level of 0.05, and assuming a standardized effect difference of 0.7% and a 20% dropout rate, we conclude that a total of 80 patients is needed to ensure statistically significant results.

### 2.8 Statistical analysis

Efficacy analysis will be conducted for the intention to treat population primarily according to the full analysis set (FAS) principle and secondarily according to the per-protocol (PP) principle. Missing values will be imputed by the last-observation-carried-forward method. Safety analysis will be conducted by FAS and PP in aggregate. The statistical analysis will be performed using SPSS. *P* < .05 is considered statistically significant.

### 2.9 Efficacy assessment

#### 2.9.1 Primary outcome

The primary outcome will be measured by SVLT-E. The primary endpoint will be the change in score from baseline to 12 weeks. For efficacy assessment, we will use the covariance analysis model that includes the SVLT-E scores as covariates and the trial centers as fixed effect variables.

#### 2.9.2 Secondary outcomes

The secondary outcomes will be measured by SVLT-E, RCFT, DST, calculation, K-CWST, DSC, K-TMT-E, COWAT, K-BNT, MMSE-DS, MoCA-K, PIT-C, EQ-5D, KQOL-AD, cost analysis, CTIS, BPPA, BIA, WED, and fNIRS. The secondary endpoints will include change in SVLT-E score from baseline to 24 weeks; change in RCFT, DST, K-BNT, calculation, COWAT, K-CWST, DSC, K-TMT-E, MMSE-DS scores from baseline to 12 weeks; change in EQ-5D, PIT-C, MoCA-K score from baseline to 6, 12, and 24 weeks; and change in KQOL-AD score from baseline to 6, 12, and 24 weeks. The cost analysis (performed at 0, 12, and 24 weeks), CTIS, BPPA, and BIA values (0, 12 weeks), and WED and fNIRS values (0, 6, 12 weeks) will also be secondary endpoints. For efficacy assessment of SVLT-E, MMSE-DS, EQ-5D, PIT-C, MoCA-K, KQOL-AD, and cost analysis variables, we will use linear mixed models that include baseline score (if there is a measured value before the intervention) as covariates and the trial centers as fixed effect variables. For efficacy assessment of RCFT, DST, K-BNT, calculation, COWAT, K-CWST, DSC, K-TMT-E variables, we will use the covariance analysis model that includes baseline score (if there is a measured value before the intervention) as covariates and the trial centers as fixed effect variables. For efficacy assessment of CTIS, BPPA, BIA, WED, and fNIRS continuous variables, we will use linear mixed models. In the case of categorical variables, we will present a contingency table and evaluate them in an exploratory way.

### 2.10 Safety assessment and adverse events report

Change in laboratory test results will be clinically evaluated. Interviews about adverse events and vital signs will be performed at every visit will be reported for safety assessment.

#### 2.10.1 Safety assessment

Safety assessment will be performed comprehensively for the FAS and PP groups. A list of adverse events including occurrence time, frequency, severity (mild, moderate, severe), and intervention causality (definitely related, probably related, possibly related, probably not related, definitely not related, unknown) will be presented, with graphs if necessary. If statistical analysis is required, the paired *t*-test, McNemar test, analysis of variance, *t*-test, Chi-square test, and/or Fisher exact test will be performed according to the characteristics of the variables and purpose of the statistical evaluation.

#### 2.10.2 Adverse events report

The investigator in charge will be required to report any adverse events during the trial to the PI within 24 hours, regardless of whether or not they are related to the intervention. In the case of a severe adverse event, the PI should stop the trial until further instruction is given, notify the client immediately, and provide an additional detailed report within 5 days. The client should promptly report to the other relevant investigators, the Institutional Review Board (IRB), and the Director of the Ministry of Food and Drug Safety.

### 2.11 Data management

Medical information obtained from this trial will be recorded in each patient's case report form (CRF) and remain confidential. After the end of the trial, a copy of the documents related to this trial including records of participants, records of drug use and administration, patient informed consent, and CRFs will be kept for 3 years in the trial center's document storage.

### 2.12 Monitoring

An independent monitoring staff will be responsible for supervising the trial process, and will periodically review and verify whether the trial is being conducted and documented in accordance with the plan, standard work guidelines, and relevant regulations. Monitoring will be performed by calling and visiting the investigator. When visiting, the monitoring staff will check the documents and from this trial, and consult the investigator if there is any problem.

### 2.13 Ethics and dissemination

This trial will be performed in accordance with the Declaration of Helsinki, ICH-GCP (International Council of Harmonization-Good Clinical Practice) guidelines, Pharmaceutical Affairs Law, and all other applicable regulations. It has been approved by the IRB of the Daejeon University Dunsan Medical Center (DJDSKH-18-DR-16) since August 31, 2018. Any protocol deviations will be approved by the IRB of the Daejeon University Dunsan Medical Center. All participants or authorized surrogates will be given a detailed explanation about this trial with the consent form and given appropriate time to determine consent or assent.

## 3 Discussion

This randomized, double-blind, placebo-controlled, parallel-group, multicenter clinical trial protocol is to evaluate the safety and efficacy of JDH compared to placebo for mNCD patients. SVLT is a standardized test in South Korea that is used to evaluate language memory, and consists of immediate recalls, delayed recalls, and a recognition test. SVLT-E is standardized version of the test that is designed especially for elderly people.^[9]^ We selected SVLT-E to be the primary outcome of this trial, as it is the most suitable version of the test for observing the progress of mNCD.

In South Korea, JDH has been used for memory deficit, but information about its effectiveness is lacking. Furthermore, there have been no clinical trials evaluating JDH to date. Although this study is limited in number of subjects, it is the first randomized controlled trial conducted with JDH. Moreover, if the hypothesis is verified, it will support wide use of JDH for the treatment of mNCD. Furthermore, we expect this trial will lead to further research on herbal medicine therapies for mNCD.

## Author contributions

**Conceptualization:** Hyung-Won Kang, In Chul Jung

**Formal analysis:** Wee-Chang Kang

**Methodology:** Jin-Hyung Jeong, Ji-yoon Lee, Yang-Chun Park, So-Jung Park, Hye-Kyoung Jang

**Supervision:** In Chul Jung

**Writing–original draft:** Jin-Hyung Jeong, Young-Kyung Seo

**Writing–review and editing:** Jin-Hyung Jeong, Ji-yoon Lee, Ju-Yeon Kim
